# MARQulS: quality improvement strategies for European cross-border healthcare

**DOI:** 10.1136/qshc.2008.032110

**Published:** 2009-01-26

**Authors:** P Vallejo, R Suñol

**Affiliations:** Avedis Donabedian University Institute–Autonomous University of Barcelona, CIBER Epidemiology and Public Health (CIBERESP), Barcelona, Spain

This supplement is focused on the methods, results and recommendations from the research project “Methods of Assessing Response to Quality Improvement Strategies” (MARQuIS). MARQuIS was funded by the Scientific Support to Policies component of the European Union Sixth Framework Research Programme (Contract SP21-CT-2004-513712), and it lasted from January 2004 to June 2008.

MARQuIS was coordinated by R Suñol, assisted by P Vallejo, from the Avedis Donabedian University Institute (Autonomous University of Barcelona) in Spain. Some of the European leading organisations in the healthcare quality field participated in this study, with two different roles:

- Eight organisations (partners) were involved in research and dissemination activities: the European Hospital and Healthcare Federation (Belgium), the European Society for Quality in Healthcare (Ireland), the Centre for Research and Advanced Training (Italy), the Department of Social Medicine, Academic Medical Centre/University of Amsterdam (The Netherlands), the Dutch Institute for Healthcare Improvement (The Netherlands), the Manchester Centre for Public Policy and Management (UK), the National Centre for Quality Assessment in Health Care (Poland) and the Avedis Donabedian University Institute (Spain).
- A country coordinator was involved in the coordination and development of the MARQuIS field test in each of the eight involved countries (except for Belgium, which had two coordinators): École de Santé Publique, Université Libre de Bruxelles and Katholieke Universiteit Leuven (Belgium), Spojena akreditacni komise Ceske Republiky (Czech Republic), Agence Nationale d’Accréditation et d’Evaluation en Santé (France), National Centre for Quality Assessment in Health Care (Poland), Fundación para la Acreditación y el Desarrollo Asistencial (Spain), Nederland’s Instituut voor Accreditatie van Ziekenhuizen (The Netherlands), The Health Quality Service (UK) and the Irish Health Services Accreditation Board (Ireland).

The aim of MARQuIS was to assess the value of different quality strategies and to provide the required information for countries when contracting care for patients moving across borders and for individual hospitals when reviewing the design of their quality strategies. Citizens can travel freely within Europe, reassured that they will have access to healthcare should an emergency arise.^1^ This research project explored whether these citizens can also be confident that the care received in another EU country will be safe and of high quality. It also explored the initiatives that could be undertaken by the European Union, the governments and the healthcare organisations to guarantee a homogeneous level of quality and safety.

The articles included in this supplement deal with known topics (quality improvement strategies, governance, patient safety, patient orientation and so on), but they are now studied in a new and evolving scenario: the increased movement of citizens and the free-market provisions of European law, enforced through a succession of judgements in the European Court of Justice, have made it difficult, or even impossible, for any one state to control and regulate its healthcare system in isolation.^2^ Interest in quality and safety at a transnational and international level is, therefore, increasing. Although some nationally initiated reforms already demonstrate some degree of policy convergence,^3^ MARQuIS identified considerable variations regarding policy directives, implementation of health-system quality initiatives and fulfilment of quality requisites between and within the countries participating in the study. This supplement provides evidence-based advice on how to promote convergence, mutual learning and coordination on these matters.

The focus of research on healthcare quality is also evolving. After many years exploring how quality could be measured, literature has shifted to explore how quality could be improved. During the last 10 or 15 years, research increasingly focused on assessing the effectiveness of quality improvement.^4^ ^5^ Effectiveness studies try to identify if quality improvement initiatives actually impact on the quality of care and which tools, mechanisms and strategies are most effective. MARQuIS fits into this research line, exploring the effectiveness of different quality improvement strategies for cross-border patients. Although this study is focused on cross-border care, cross-border patients are merely citizens with a condition for which they require healthcare and who happen to receive that care in a place that is different to their country of origin. Quality of care for cross-border patients, as covered in this study, other than the specific requirements and demands from foreign patients, also covers all the dimensions that affect quality during any care process: patient safety, patient centredness, clinical effectiveness, efficiency, staff orientation and responsive governance.^6^ For this reason, most of the results and recommendations from this study can be applied to any healthcare organisation, regardless of their cross-border population. We hope that those working in the field of healthcare (healthcare professionals and managers, researchers and healthcare policy makers at national and transnational levels) will be able to use the ideas, methods and recommendations from this research.

The first article of this supplement (by Suñol *et al*^7^) contextualises the research project, analysing the legal context, regulations and experiences of cross-border care in Europe. The next two articles (Vallejo *et al*^8^ and Groene *et al*^9^) explore the nature of the European cross-border care phenomenon by identifying the quantity and type of care provided and the specific quality requirements for this population. Spencer and Walshe^10^ identified the most relevant national quality improvement policies and strategies in Europe. The results of these studies, performed in the first phases of the project, were the basis for developing the questionnaire and audit tools used to gather information from 389 hospitals in eight EU countries during the field test. The remaining articles in this supplement are based on the analysis of the information collected during the field test, describing the implementation of quality improvement strategies (Lombarts *et al*^11^), safety mechanisms (Suñol *et al*^12^) and governance (Shaw *et al*^13^) in European hospitals, and exploring the relation between the implementation of quality-improvement strategies and the fulfilment of patient-centredness requirements (Groene *et al*^14^) and the fulfilment of hospital outputs (Suñol *et al*^15^). The paper by Lombarts *et al*^16^ presents a new classification scheme that might be a first step towards a generic quality improvement-maturity instrument, which could then be used as a practical “quick scan” for assessing the maturity of a hospital’s quality improvement system. Finally, the last article^17^ of this supplement presents the recommendations to different stakeholders based on the results of this study and their implications for future action in policy and research.

MARQuIS, as well as other recent studies on this topic, such as Europe for Patients and SIMPATIE, faced similar challenges in study design and development, mainly due to differences in the organisation of healthcare systems, the information available on healthcare databases and the use of different languages and terminologies between Member States. Even with these limitations, one of the main achievements of these projects is their contribution to placing the topics of quality of care and patient safety high on the European Agenda. The European Commission’s Seventh Framework research programme contains a number of initiatives focused on optimising the delivery of healthcare to European citizens, and the recent proposal for a directive on the rights of patients to cross-border care^18^ explicitly highlights the importance of effective mechanisms for ensuring quality and safety of healthcare. Future action in the field of European quality and safety is thus to be expected. We therefore encourage healthcare researchers to further continue the research on the effectiveness of quality-improvement strategies and mechanisms, both for cross-border patients and for the general population, based on MARQuIS and other relevant research.
